# Quantitative measurement of thyroglobulin mRNA in peripheral blood of patients after total thyroidectomy

**DOI:** 10.1054/bjoc.2001.1904

**Published:** 2001-07

**Authors:** T Takano, A Miyauchi, H Yoshida, Y Hasegawa, K Kuma, N Amino

**Affiliations:** Department of Laboratory Medicine, Osaka University Medical School, D2, 2-2 Yamadaoka, Suita, Osaka, 565-0871; Kuma Hospital, 8-2-35, Simoyamatedori, Chuo-ku, Kobe, Hyogo 650-0011, Japan

**Keywords:** thyroglobulin, real-time quantitative RT-PCR, thyroid cancer, molecular-based diagnosis

## Abstract

Previous studies have reported the clinical usefulness of reverse transcription-polymerase chain reaction (RT-PCR) detection of thyroglobulin (TG) mRNA in the peripheral blood of patients with differentiated thyroid carcinoma. To evaluate this usefulness, we measured TG mRNA in the peripheral blood of patients diagnosed with thyroid carcinoma after total thyroidectomy by real-time quantitative RT-PCR using glyceraldehyde-3-phosphate dehydrogenase (GAPDH) mRNA as an internal control. Surprisingly, we detected TG mRNA in all samples obtained after total thyroidectomy, including those from 4 medullary carcinomas. Further, there was no statistical difference in expression levels of TG mRNA in the patients with or without metastasis, and no significant correlation was found between serum TG concentrations and the expression levels of TG mRNA. These results give rise to a question regarding the clinical applications of not only RT-PCR detection but also quantitative measurement of TG mRNA in peripheral blood. © 2001 Cancer Research Campaign http://www.bjcancer.com


*British Journal of Cancer* (2001) **85**(1), 102–106
© 2001 Cancer Research Campaign
doi: 10.1054/ bjoc.2001.1904, available online at http://www.idealibrary.com on IDEAL®

### Quantitative measurement of thyroglobulin mRNA in peripheral blood of patients after total thyroidectomy

**T Takano[1], A Miyauchi[2], H Yoshida[2], Y Hasegawa[1], K Kuma[2] and N Amino[1]**

[1]Department of Laboratory Medicine, Osaka University Medical School, D2, 2-2 Yamadaoka, Suita, Osaka 565-0871; [2]Kuma Hospital, 8-2-35, Simoyamatedori, Chuo-ku, Kobe, Hyogo 650-0011, Japan

**Summary** Previous studies have reported the clinical usefulness of reverse transcription-polymerase chain reaction (RT-PCR) detection of thyroglobulin (TG) mRNA in the peripheral blood of patients with differentiated thyroid carcinoma. To evaluate this usefulness, we measured TG mRNA in the peripheral blood of patients diagnosed with thyroid carcinoma after total thyroidectomy by real-time quantitative RT-PCR using glyceraldehyde-3-phosphate dehydrogenase (GAPDH) mRNA as an internal control. Surprisingly, we detected TG mRNA in all samples obtained after total thyroidectomy, including those from 4 medullary carcinomas. Further, there was no statistical difference in expression levels of TG mRNA in the patients with or without metastasis, and no significant correlation was found between serum TG concentrations and the expression levels of TG mRNA. These results give rise to a question regarding the clinical applications of not only RT-PCR detection but also quantitative measurement of TG mRNA in peripheral blood. © 2001 Cancer Research Campaign http://www.bjcancer.com

**Keywords**: thyroglobulin; real-time quantitative RT-PCR; thyroid cancer; molecular-based diagnosis

Thyroid carcinomas often recur many years after surgery (Loh et al, 1997). The monitoring of serum thyroglobulin (TG) by immunoassay can be used in detecting residual or recurrent differentiated thyroid carcinoma (DTC) after total thyroidectomy. However, the usefulness of this method is limited by both the requirement for thyroid hormone withdrawal to attain optimal sensitivity and interference by antithyroglobulin antibodies (Singer et al, 1996). Recent reports have demonstrated that the reverse transcription-polymerase chain reaction (RT-PCR) can be used to detect circulating cancer cells in the peripheral blood of patients with malignancies such as prostate cancer (Ghossein et al, 1995) and neuroblastoma (Mattano et al, 1992).

A sensitive RT-PCR assay amplifying thyroid-specific mRNAs such as TG or thyroid peroxidase (TPO) may be utilized for the early detection of DTC recurrence and thus may have important therapeutic and prognostic implications. In fact, 2 recent reports have shown the clinical usefulness of the RT-PCR detection of TG, TPO, and ret/PTC in the follow-up of DTC (Ringel et al, 1998; Tallini et al, 1998). For example, TG mRNA in peripheral blood became detectable earlier than serum TG in a case of recurrent thyroid papillary carcinoma. Recently, Ringel et al have reported the clinical usefulness of the real-time quantitative measurement of TG mRNA in the peripheral blood of the patients with DTC. In their report, however, they did not use an appropriated internal reference such as glyceraldehyde-3-phosphate dehydrogenase (GAPDH) mRNA (Ringel et al, 1999).

Encouraged by these optimistic reports, we applied this method to our patients for the follow-up of DTC. However, we found that TG and TPO mRNAs were detectable in the peripheral blood of all the patients tested, a result that differs from those reported previously, and we therefore felt a need to re-evaluate TG mRNA detection. One of the problems of previous studies has been that the expressions levels of TG mRNA have not been calculated in conjunction with the use of an appropriate internal control, as the results of simple RT-PCR detection can vary in response to subtle changes in conditions.

Patients who have undergone total thyroidectomy constitute good model cases for evaluating this problem. As such, we measured TG mRNA in the peripheral blood of patients after total thyroidectomy and determined the expression levels of TG mRNA by real time-quantitative RT-PCR using GAPDH mRNA as an internal control.

*Received 22 June 2000*
*Revised 23 April 2001*
*Accepted 23 April 2001*

*Correspondence to:* T Takano

### SUBJECTS AND METHODS

### Patients

We evaluated 57 patients (3 males and 54 females, between 15 and 77 years of age, 49 papillary, 4 follicular, and 4 medullary carcinomas) who underwent total thyroidectomy. Blood was drawn at least 6 months after surgery or the administration of radioactive iodine. Of the 53 DTC patients, 21 had evident metastases (16 lung, 1 bone, 2 mediastinum, and 2 lung and bone) detected by either computed tomography or $^{131}$I scintigram. All the patients with metastasis and 21 of 32 patients without metastasis received 5 to 150 (mean: 55.7 mCi) and 5 to 100 (mean: 17.0 mCi) $^{131}$I, respectively. All the patients after total thyroidectomy, except those with medullary carcinoma, received a suppressive dose of thyroxine so that their serum thyroid stimulating hormone (TSH) levels were undetectable. 17 healthy subjects (5 males and 12 females, between 29 and 59 years of age) with no evidence of thyroid disease were also examined as controls. The protocol was approved by the institutional review boards at the participating institutions, and informed consent was obtained.

### Blood samples and RNA preparation

Peripheral blood was collected in heparinized tubes in 10 ml samples and placed immediately on ice. Next, 10 ml of 3% dextran 200 000 (WAKO, Osaka, Japan) was added to the tubes, which were than placed in ice for 30 min. The supernatant was collected and centrifuged. The precipitant was dispersed in distilled water to lyse the remaining erythrocytes, and an equal volume of 1.8% NaCl was then immediately added. The isolated cells were washed, then centrifuged. Total RNA was extracted following standard procedures (Chomczynski and Sacchi, 1987) and resolved in 20 μl of distilled water, then stored at –70˚C.

### Reverse transcription

1 μl of total RNA was reverse transcribed in an RT mixture containing 50 mM Tris-HCl (pH 8.3), 75 mM KCl, 10 mM dithiothreitol, 3 mM $MgCl_2$, 0.5 mM deoxynucleotide triphosphates (dNTPs), 200 U M-MLV reverse transcriptase (Gibco, Gaithersburg, MD), 2 U $\mu l^{-1}$ RNase inhibitor (Takara, Shiga, Japan), and 2.5 μM random hexamer (Takara) in a total volume of 20 μl at 42˚C for 60 min.

### RT-PCR detection of TG mRNA

1 μl of first-strand cDNA was used as a template for the PCR reaction with specific primers for TG cDNA as previously described (Tallini et al, 1998). Each reaction mixture consisted of 1 μl of cDNA, 0.5 μM of each primer, 1 μl of 10 × Ex *Taq* Buffer, 0.8 μl of 2.5 mM dNTP mix, 0.5 U of Ex *Taq* polymerase, and nuclease-free water to a final volume of 10 μl. 10 × Ex *Taq* Buffer, dNTP mix, and Ex *Taq* polymerase were obtained from Takara. The reaction mixture was then subjected to 35 cycles of denaturation (94˚C, 1 min), annealing (55˚C, 1 min) and extension (72˚C, 1 min). After PCR amplification, the reaction mixture was run on a 2% SeaKem GTG agarose gel (Takara). The gel was then stained with ethidium bromide. Samples without reverse transcription were used as negative controls.

### Real-time quantitative RT-PCR

Real-time quantitative RT-PCR (TaqMan PCR) of TG and GAPDH mRNAs using an ABI PRISM 7700 Sequence Detection System and a TaqMan PCR Core Reagent Kit (PE Biosystems, Foster City, CA) was performed as previously described (Takano et al, 1999). The primers used for amplification of TG cDNA were changed to the same set of primers used in the report by Tallini et al. The probe for TG cDNA used for the TaqMan PCR was: (5′-FAM-CACTTCGAGTTCCAGGAATGGCCTGACCCT-TAMRA-3′).

1 μl of each cDNA was used for measurement of the copy number. The conditions for the TaqMan PCR were as follows: 95˚C for 10 min, followed by 40 cycles of 95˚C for 15 seconds and 60˚C for 1 min. Recombinant pGEM T-vectors (Promega, Tokyo, Japan) containing TG or GAPDH cDNA were constructed by PCR cloning with the same set of primers used in the TaqMan PCR and were used as standard samples. The expression levels of TG mRNA were calculated by dividing the copy number of TG mRNA by that of GAPDH mRNA. Intraassay variabilities were 35% and 24% at 10 and 100 ng $l^{-1}$ thyroid total RNA, respectively. Interassay variabilities across a 1-month period were 38% and 28% at 10 and 100 ng/L thyroid total RNA, respectively.

### Extraction of total RNA from a normal thyroid tissue

A portion of normal thyroid tissue from the opposite lobe of a thyroid papillary carcinoma was obtained by surgery. Total RNA was extracted following standard procedures.

### Serum TG measurement

Serum TG was measured using a commercial radioimmunoassay kit (Ab-Beads Thyroglobulin, Eiken, Tokyo, Japan). Intrassay variability was 8.6% at 5 $\mu g\ l^{-1}$. Interassay variabilities across a 1-month period were 11.8%, 7.4%, 3.1% at 15, 15, 50 $\mu g\ l^{-1}$, respectively. Assay sensitivity as determined from the 20% interassay coefficient of variation (CV) was 0.5 $\mu g\ l^{-1}$. The cut off level of this kit was 28.4 $\mu g\ l^{-1}$. Serum anti-TG antibody was detected with a semi-quantitative microtitre particle agglutination test for the in vitro diagnostic detection and titration of anti-TG antibodies in human serum, SERODIA-ATG (Fujirebio, Tokyo, Japan).

### Statistical analysis

Statistical analysis of differences between the groups was carried out using the Mann–Whitney *U* test. Linear correlation analysis was used to examine the correlation between serum TG and expression levels of TG mRNA in the peripheral blood. *P*s of $<0.05$ were considered significant.

### RESULTS

TG mRNA was detected in all the samples tested, including the samples from 4 patients diagnosed with medullary carcinoma. Representative data are shown in Figure 1.

The reliability of the quantitative RT-PCR was estimated by adding total RNA from a normal thyroid to that from 10 ml of blood of a healthy subject. The TG/GAPDH mRNA ratio in the sample without the addition of thyroid RNA was $6.75 \times 10^{-5}$. As the thyroid RNA was increased from 10 ng per 1 l blood to 10 μg per 1 l blood, a linear increase in the TG/GAPDH mRNA ratio was observed, indicating the reliability of this quantitative measurement (Figure 2). These results were used in the construction of the standard curve in the following experiments.

Using this method, the expression levels of TG mRNA in the peripheral blood of 57 patients and 17 healthy subjects were calculated. All the samples were successfully measured by real-time quantitative RT-PCR, which confirmed the expression of TG mRNA in all the samples. There was no statistical difference between the patients with and without metastasis (Figure 3). The serum TG was measured in the 49 DTC patients without serum anti-TG antibody, and the set of results were compared (Figure 4). No statistical differences in the TG mRNA levels were observed. While serum TG was detectable in all the patients with metastasis, it was undetectable in 14 of 29 patients without metastasis. Further, serum TG was undetectable in all 4 patients diagnosed with medullary carcinoma, while quantitative measurement of TG mRNA was possible (Figure 3). These results suggest that serum TG is a superior marker of distant metastasis of DTC than TG mRNA in peripheral blood. Further, the correlation between TG

© 2001 Cancer Research Campaign

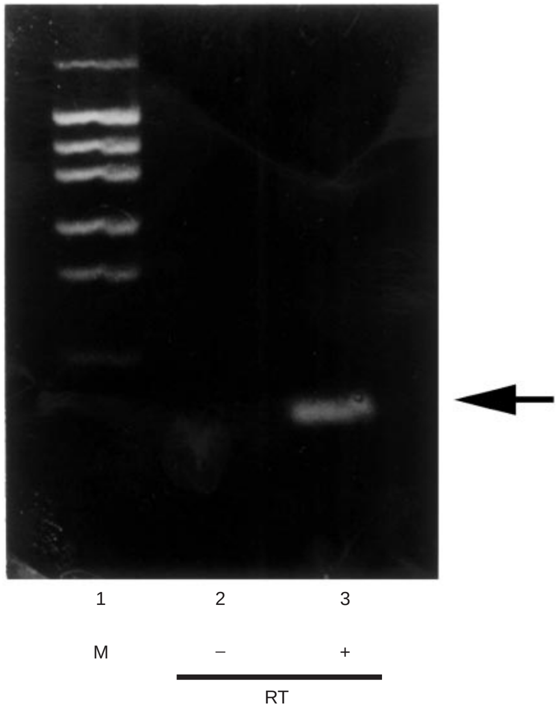

**Figure 1** Expression of TG mRNA in the peripheral blood of a patient diagnosed with medullary carcinoma after total thyroidectomy. After extraction of total RNA from the peripheral blood, RT-PCR amplifying TG cDNA was performed (Lane 3). Lane 2 shows the results using the same sample without the addition of reverse transcriptase. Lane 1; PHY maker (Takara)

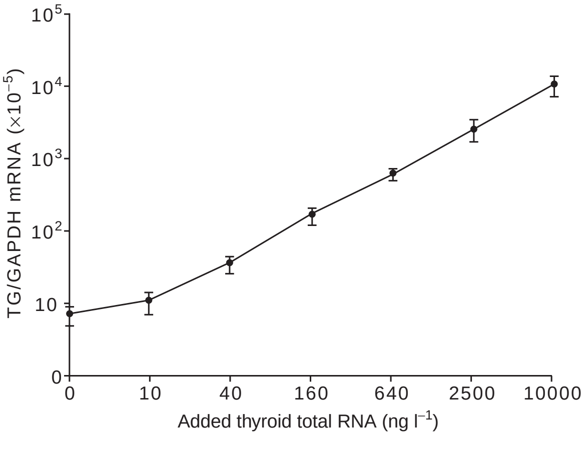

**Figure 2** Real-time quantitative RT-PCR of TG mRNA in peripheral blood. Total RNA from a normal thyroid was added to that from 10 ml of blood from a healthy subject, after which real-time quantitative RT-PCR, as described in Methods was carried out. The copy number of GAPDH mRNA was simultaneously measured, and the expression levels of TG mRNA were calculated as the ratio of TG and GAPDH mRNA. The results are shown with mean ±SD for triplicate determinations mRNA and serum TG was estimated in these 49 patients and no correlation was observed between the two (Figure 5).

### DISCUSSION

The vast majority of thyroid carcinomas are differentiated tumours that originate from the follicular epithelium and show

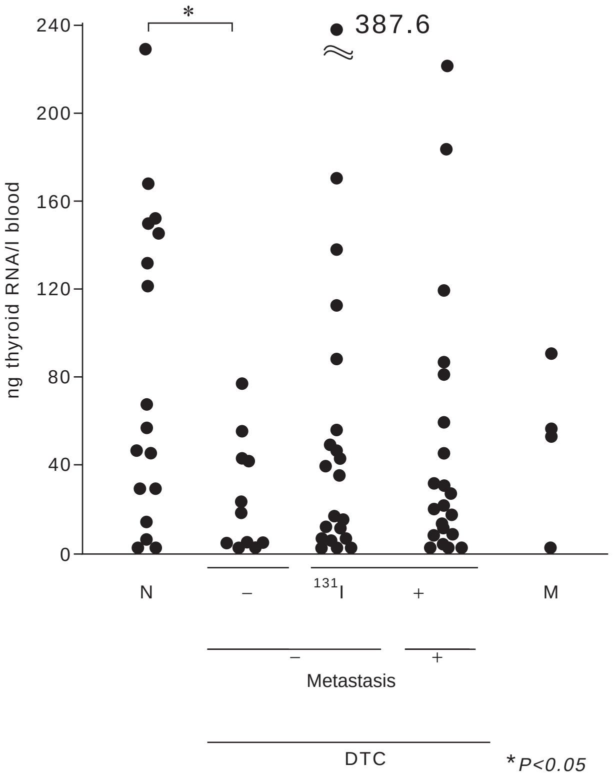

**Figure 3** TG mRNA in peripheral blood of the patients after total thyroidectomy. The expression levels of TG mRNA in the peripheral blood of healthy subjects (N), the patients of DTC after total thyroidectomy (DTC) with or without distant metastasis, and patients of medullary carcinoma (M) were measured. The results are shown with means for duplicate determinations

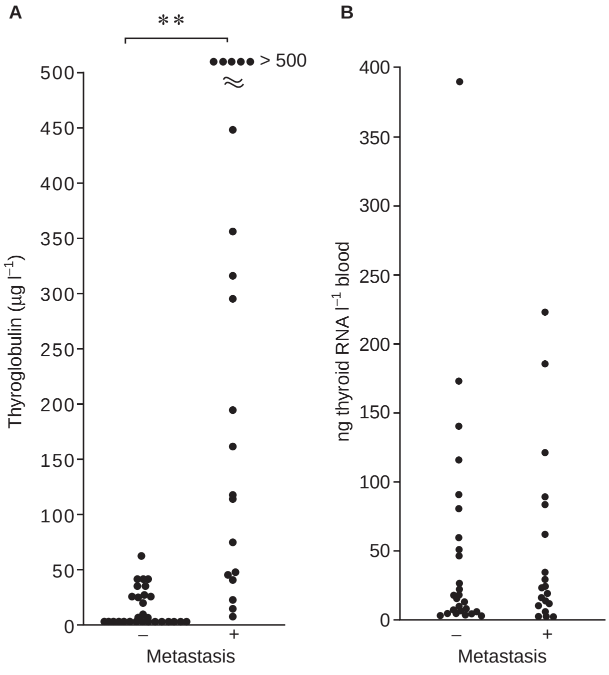

**Figure 4** Comparison of serum TG (**A**) and TG mRNA (**B**) in the patients with (+) or without (–) metastasis

*© 2001 Cancer Research Campaign*

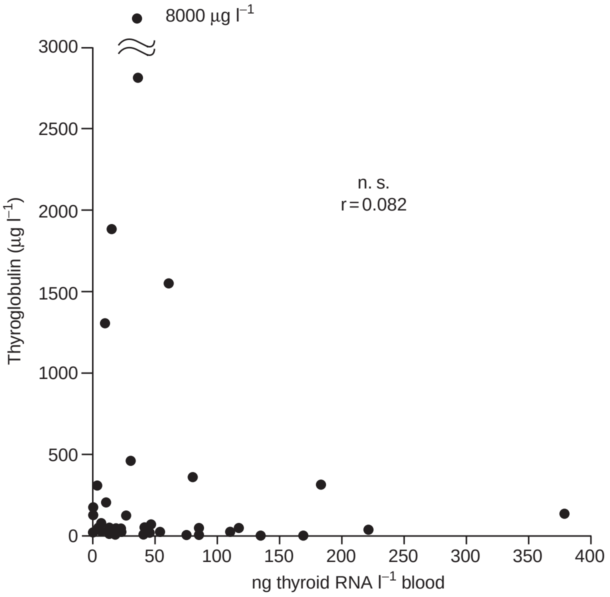

**Figure 5** Correlation between the expression levels of TG mRNA and the serum TG concentrations either follicular or papillary structure. Serum TG immunodetection measurements are currently the best available indicator of tumour metastases, even though their diagnostic usefulness has several limitations.

Circulating tumour cells can be detected by sensitive PCR amplification of tumour-specific abnormal gene sequences or through the detection of tissue-specific abnormal gene transcripts normally absent in the peripheral blood. Thyroid tumours represent the ideal target for such studies because they actively express tissue-specific markers such as TG and TPO. Despite promising reports in 1998 on the detection of TG mRNA in peripheral blood, our preliminary study showed that TG transcripts were detectable in all samples tested, including the ones from healthy subjects. Recently, 2 studies suggesting limitations in the clinical applications of this method have been reported. Wingo et al have detected TG transcripts in the peripheral blood of healthy subjects that can be measured by real-time quantitative RT-PCR (Wingo et al, 1999). Bojunga et al have found that TG mRNA expression is not specific to thyroid tissue and is not correlated with a diagnosis of thyroid cancer in patients, and they have recommended further examination of this method by quantitative measurement (Bojunga et al, 2000). We therefore decided to re-evaluate this method using samples obtained from patients after total thyroidectomy, in which TG transcripts in the circulating thyroid cells from the normal thyroid may be negligible. We detected TG transcripts in all the samples tested, even in that of the patient diagnosed with medullary carcinomas, in whom the circulating of thyroid follicular cells was not likely to occur. Real-time quantitative RT-PCR assay confirmed the expression of TG mRNA in these samples, in which an increase in the fluorescent signal from hybridized TG cDNA-specific probe was observed.

By real-time quantitative RT-PCR, high levels of TG mRNA were expressed in some patients without evident metastasis. For example, in one patient, the copy number of TG mRNA in 1 ml of peripheral blood was comparable to that in over 300 pg total RNA from normal thyroid tissue, which is approximately equal to 30 thyroid cells. It is hard to believe that this many thyroid cells still circulated in the peripheral blood of a patient after thyroidectomy without any evidence of a distant metastasis, which could be a source of circulating cells. TSH receptor mRNA, which has previously been considered to be expressed only in the thyroid, was found to be expressed in adipocytes and lymphocytes (Francis et al, 1991; Endo et al, 1995). The TG mRNA detected by our study thus might not have been derived from thyroid cells; instead, it is most likely that lymphocytes express small quantities of TG mRNA, as they are known to express various kinds of mRNAs such as alpha-fetoprotein (AFP) or carcinoembryonic antigen (CEA) (Lafarge-Frayssinet et al, 1989; Coutelier et al, 1994), which used to be considered tumour-specific. Further, the expression of TG mRNA is not likely to be limited to thyroid follicular cells, as we have recently found it to be expressed in cell lines derived from lung or gastric carcinomas (data not shown). Possibly, the regulation of TG mRNA expression in lymphocytes is controlled by some unknown factors. If so, the detection of TG mRNA is not likely to become an alternative means of achieving early detection of recurrent thyroid carcinomas because sensitive detection of recurrence would be interfered with by basal expression of TG mRNA in peripheral blood.

It therefore seems likely that the diagnostic methods presented by Tallini and Ringel may be useful only in limited populations of patients in whom a considerable number of thyroid tumour cells expressing a high copy number of TG mRNA are circulating. In fact, our results showed no statistical difference between the samples from patients with or without distant metastasis. Further, we obtained no evidence that this method is superior to the conventional measurement of serum TG, as it was hard to utilize the TG mRNA data to differentiate the patients with metastasis from those without, whereas serum TG measurements showed more than 5 μg $l^{-1}$ in all patients with distant metastasis, and the high values of interassay CV in the quantitative measurement of TG mRNA can be a disadvantage in the follow-up of a patient for a long period. Moreover, TG mRNA and serum TG measured in the same samples showed no significant correlation. These results suggest that the sources of TG mRNA and serum TG might be different, and, as discussed above, the former might not be derived from thyroid follicular cells.

The use of TPO mRNA in the detection of thyroid carcinomas has also been reported in conjunction with some studies. However, our preliminary data showed that TPO mRNA was detectable by RT-PCR in all the samples tested, including those from healthy subjects and patients after total thyroidectomy without distant metastasis (data not shown). Considering that TPO mRNA are usually less abundant than TG mRNA in thyroid cells, it is not likely that the use of TPO mRNA instead of TG would improve the clinical usefulness of this method.

In summary, we have found no advantages in diagnosing DTC by the quantitative measurement of TG mRNA in peripheral blood. We consider that an intensive re-evaluation, including a determination of what percentage of TG mRNA derives from thyroid follicular or cancer cells, is necessary with regard to this method before considering the clinical applications.

### ACKNOWLEDGEMENT

This work was supported by a Grant-in-Aid for Encouragement of Young Scientists (to TT; No. 12771474) from the Ministry of Education, Science, Sports and Culture of Japan.

© 2001 Cancer Research Campaign

### REFERENCES

Bojunga J, Roddiger S, Stanisch M, Kusterer K, Kurek R, Renneberg H, Adams S, Lindhorst E, Usadel KH and Schumm-Draeger PM (2000) Molecular detection of thyroglobulin mRNA transcripts in peripheral blood of patients with thyroid disease by RT-PCR. *Br J Cancer* **82**: 1650–1655

Chomczynski P and Sacchi N (1987) Single-step method of RNA isolation by acid guanidinium thiocyanate-phenol-chloroform extraction. *Anal Biochem* **162**: 156–159

Coutelier JP, Godfraind C, Dveksler GS, Wysocka M, Cardellichio CB, Noel H and Holmes KV (1994) B lymphocyte and macrophage expression of carcinoembryonic antigen-related adhesion molecules that serve as receptors for murine coronavirus. *Eur J Immunol* **24**: 1383–1390

Endo T, Ohta K, Haraguchi K and Onaya T (1995) Cloning and functional expression of a thyrotropin receptor cDNA from rat fat cells. *J Biol Chem* **270**: 10833–10837

Francis T, Burch HB, Cai WY, Lukes Y, Peele M, Carr FE, Wartofsky L and Burman KD (1991) Lymphocytes express thyrotropin receptor-specific mRNA as detected by the PCR technique. *Thyroid* **1**: 223–228

Ghossein RA, Scher HI, Gerald WL, Kelly WK, Curley T, Amsterdam A, Zhang ZF and Rosai J (1995) Detection of circulating tumor cells in patients with localized and metastatic prostatic carcinoma: clinical implications. *J Clin Oncol* **13**: 1195–200

Lafarge-Frayssinet C, Torres JM, Frain M and Uriel J (1989) Alpha-fetoprotein gene expression in human lymphoblastoid cells and in PHA-stimulated normal T-lymphocytes. *Biochem Biophys Res Commun* **159**: 112–118

Loh KC, Greenspan FS, Gee L, Miller TR and Yeo PP (1997) Pathological tumor-node-metastasis (pTNM) staging for papillary and follicular thyroid carcinomas: a retrospective analysis of 700 patients. *J Clin Endocrinol Metab* **82**: 3553–3562

Mattano LA, Jr., Moss TJ and Emerson SG (1992) Sensitive detection of rare circulating neuroblastoma cells by the reverse transcriptase-polymerase chain reaction. *Cancer Res* **52**: 4701–4705

Ringel MD, Ladenson PW and Levine MA (1998) Molecular diagnosis of residual and recurrent thyroid cancer by amplification of thyroglobulin messenger ribonucleic acid in peripheral blood. *J Clin Endocrinol Metab* **83**: 4435–4442

Ringel MD, Balducci-Silano PL, Anderson JS, Spencer CA, Silverman J, Sparling YH, Francis GL, Burman KD, Wartofsky L, Ladenson PW, Levine MA and Tuttle RM (1999) Quantitative reverse transcription-polymerase chain reaction of circulating thyroglobulin messenger ribonucleic acid for monitoring patients with thyroid carcinoma. *J Clin Endocrinol Metab* **84**: 4037–4042

Singer PA, Cooper DS, Daniels GH, Ladenson PW, Greenspan FS, Levy EG, Braverman LE, Clark OH, McDougall IR, Ain KV and Dorfman SG (1996) Treatment guidelines for patients with thyroid nodules and well-differentiated thyroid cancer. American Thyroid Association. *Arch Intern Med* **156**: 2165–2172

Takano T, Miyauchi A, Yokozawa T, Matsuzuka F, Maeda I, Kuma K and Amino N (1999) Preoperative diagnosis of thyroid papillary and anaplastic carcinomas by real-time quantitative reverse transcription-polymerase chain reaction of oncofetal fibronectin messenger RNA. *Cancer Res* **59**: 4542–4545

Tallini G, Ghossein RA, Emanuel J, Gill J, Kinder B, Dimich AB, Costa J, Robbins R, Burrow GN and Rosai J (1998) Detection of thyroglobulin, thyroid peroxidase, and RET/PTC1 mRNA transcripts in the peripheral blood of patients with thyroid disease. *J Clin Oncol* **16**: 1158–1166

Wingo ST, Ringel MD, Anderson JS, Patel AD, Lukes YD, Djuh YY, Solomon B, Nicholson D, Balducci-Silano PL, Levine MA, Francis GL and Tuttle RM (1999) Quantitative reverse transcription-PCR measurement of thyroglobulin mRNA in peripheral blood of healthy subjects. *Clin Chem* **45**: 785–789

*© 2001 Cancer Research Campaign*
